# A Vascular Quartet: Scalp Arteriovenous Malformation, Sinus Pericranii, Dural Arteriovenous Fistula, and Arteriovenous Brain Malformation in a Single Patient

**DOI:** 10.7759/cureus.37140

**Published:** 2023-04-04

**Authors:** Anand Kaul, Srinivasa P Kanuparthi, Kadir Erkmen

**Affiliations:** 1 Neurosurgery, Temple University Hospital, Philadelphia, USA

**Keywords:** sinus pericranii, arteriovenous malformation, targeted transvenous embolization, transarterial embolization, endovascular coil embolization, arteriovenous (av) fistula

## Abstract

We present a case of a 51-year-old female who presented for evaluation of a large scalp mass found to have a different quartet of vascular malformations- a persistent scalp arteriovenous malformation (sAVM) with sinus pericranii, an inoperable intracranial SM-V brain arteriovenous malformation (bAVM), and a Cognard I dural arteriovenous fistula (dAVF). This is the first reported instance with four distinct vascular pathologies. We review the etiologies of multiple vascular abnormalities in the cerebral circulation that could contribute to this patient's findings and review strategies for treatment. We conducted a retrospective review of the clinical and angiographic records for a single adult female patient, including a management approach and an in-depth literature review. Given the high baseline vascularity of these complex lesions, surgery was not considered the initial therapy. We focused primarily on the sAVM with a staged embolization involving both transarterial and transvenous approaches. Transarterial coil embolizes 5 feeding artery branches of the right external carotid artery, followed by transvenous coil embolization into the common venous pouch accessed through the transosseous sinus pericranii via the SSS, dramatically reduced the size and filling of the large sAVM and eliminated a significant source of hypertensive venous outflow. Serial endovascular treatments of her sAVM led to a significant reduction in size and pulsatility, and the pain from tenderness to palpation was concurrently decreased. Despite multiple treatments, serial angiographic evaluations of her scalp lesion showed continued new development of collaterals. Ultimately the patient elected to forego further treatment for her sAVM.

To our knowledge, there has not been another report of a single adult patient with a quartet of vascular malformations in the literature. Treatment paradigms for sAVMs are limited to case reports and small series; however, we purport that the most successful therapeutic approaches are multimodal and likely should incorporate surgical resection when feasible. We emphasize the caution required for patients with multiple other underlying intracranial vascular malformations. The altered intracranial flow dynamics can drastically hinder the success of a unimodal approach involving endovascular therapy alone.

## Introduction

Due to their rarity, scalp arteriovenous malformations (sAVM) escape a robust classification scheme [1,2]. These dilated abnormal vessels are identified within the subcutaneous space and can be cosmetically disfiguring secondary to their size and the overlying skin necrosis. These lesions are prone to episodic bleeding due to their flow architecture and thus often exhibit poor wound healing. Management of sAVMs is confined mainly to case reports and small series predominantly within the pediatric population [1,3,4]. Moreover, there needs to be more regarding treating these complex vascular lesions in adults within the context of multiple distinct cohabitating vascular malformations in a single patient. Heritable causes of multiple vascular malformations are documented in the literature as part of named syndromes. These patients, however, generally develop multiple vascular lesions of a single type [5,6]. We review the etiologies of multiple vascular abnormalities in cerebral circulation and the mechanisms contributing to finding a different quartet of vascular malformations in a single adult patient. We discuss our management approach as a resource for treating other patients in similar situations, highlighting the importance of triaged therapy.

## Case presentation

Clinical presentation

A 51-year-old female presented to our institution to evaluate a large pulsating scalp mass on the patient's forehead, which was found to be a scalp AVM. Workup also revealed the large sAVM was associated with sinus pericranii, a large Spetzler-Martin (SM) grade V brain AVM (bAVM), as well as a Cognard grade I dural arteriovenous fistula (dAVF). Her scalp mass and brain AVM were discovered in childhood after an initial bleeding episode from her forehead. She was recommended for conservative management of both lesions. When she presented, she had noticed progressive enlargement of her right-sided frontal scalp AVM. Approximately five years before her presentation to our institution, she had a right frontotemporal intracranial hemorrhage prompting reimaging and subtotal endovascular embolization of the complicated bAVM nidus. She underwent multiple endovascular treatments over one year, which unfortunately culminated in an iatrogenic stoke producing left-sided hemiparesis and homonymous hemianopsia, which improved significantly with intensive rehabilitation. Notably, serial angiograms revealed a marked progression of the sAVM.

The patient had no other significant past medical history and was a non-smoker. On examination, she had mild dysarthria and slurred speech with left-sided homonymous hemianopsia, relative left-sided hemiparesis graded 4/5, with mild residual paresthesias and numbness in the left hand. She was ambulatory independently without the need for assistive devices. A large purple pulsatile lesion was noted in the region of her right forehead. The overlying skin was noted to be taut and tender to palpation with an overlying scab secondary to the multiple times it had bled and healed in recent history.

Angiographic results

**Figure 1:**
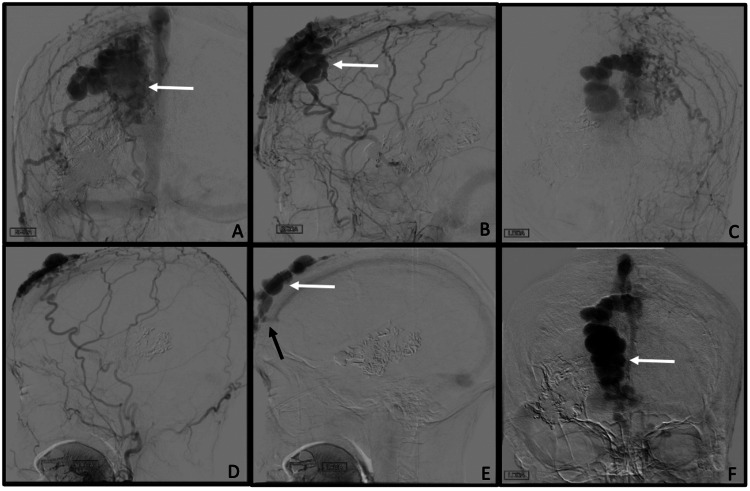
Scalp AVM and Sinus Pericranii Digital subtraction angiogram, selective right (A, B) and left (C-F) external carotid artery injections. A and B demonstrate the large venous varix measuring 8 cm x 6 cm x 4m (white arrow) and numerous arterial feeders, the largest of which is a hypertrophied superficial temporal artery. C-F Demonstrates early arterial phase demonstrating the filling of a significant scalp AVM. Arterial and venous phases are provided to demonstrate flow and drainage. The scalp AVM drains intracranially through the skull into the SSS and extracranially bilaterally through multiple facial veins. The transosseous channel indicates a sinus pericranii (black arrow) with the anterograde flow within the SSS. AVM: Arteriovenous malformations; SSS: superior sagittal sinus

Diagnostic digital subtraction angiography (DSA) via a transfemoral approach was performed under general anesthesia. The right external carotid artery showed hypertrophy of the superficial temporal artery, middle meningeal artery, and various branches supplying the scalp. The scalp AVM was identified, receiving input primarily from the right superficial temporal artery and possessing a large venous varix measuring approximately 8 cm x 6cm x 4 cm in greatest dimensions with drainage into superficial facial veins as well as intracranial drainage into the superior sagittal sinus (SSS) via a transosseous channel indicating the presence of a sinus pericranii (Figure 1).

**Figure 2:**
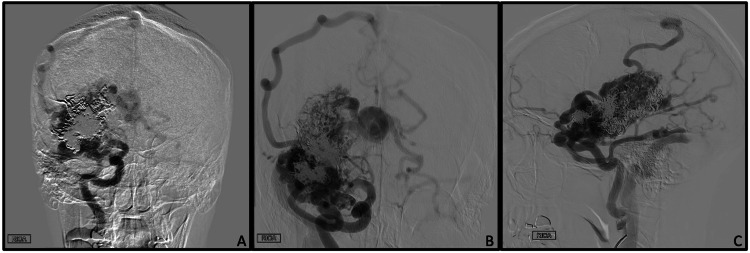
Brain Arteriovenous Malformation Selective right internal carotid artery injection with unsubtracted AP (A), subtracted AP (B), and lateral (C) projections demonstrating a large, complex Spetzler-Martin grade V AVM. Note the significant Onyx cast and residual filling of the AVM nidus with both superficial and deep venous drainage. AVM: Arteriovenous malformations

**Figure 3:**
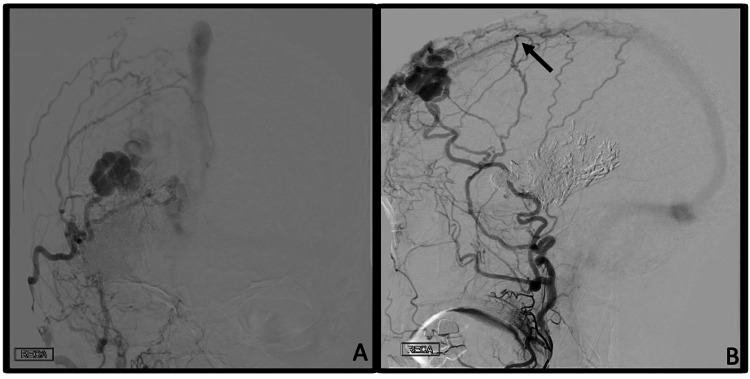
Dural AV Fistula Digital subtraction angiogram, selective right external carotid artery injection, AP (A) and lateral (B) projections, showing a Cognard type I dAVF receiving supply from right middle meningeal arterial feeders (black arrows) with anterograde drainage into the SSS. dAVF: dural arteriovenous fistula; SSS: superior sagittal sinus

Injection of the right internal carotid artery revealed the SM-V bAVM within the right frontotemporal lobe again (figure 2). Arterial supply to nidus was from the right middle cerebral artery and right posterior cerebral artery. Venous drainage of the nidus was noted via the superficial Sylvian vein down to the transverse-sigmoid junction, with an enlarged anastomotic vein of Trolard providing outflow to the SSS. Deep venous drainage was noted into hypertrophied internal cerebral veins. Significant Onyx casting was noted from her prior treatments. There was no evidence of venous outflow obstruction, intranidal aneurysm, or feeding artery aneurysm. The large AVM nidus created a steal phenomenon for the right-sided intracranial circulation; thus, the right middle cerebral artery circulation was supplied through leptomeningeal collaterals from the right ACA distribution, which filled through a patent anterior communicating artery best visualized from the left internal carotid artery injection. Lastly, a separate right-sided Cognard type I dAVF was newly identified as receiving middle meningeal branches with a terminus in the SSS (Figure 3).

Management of the scalp AVM

**Figure 4:**
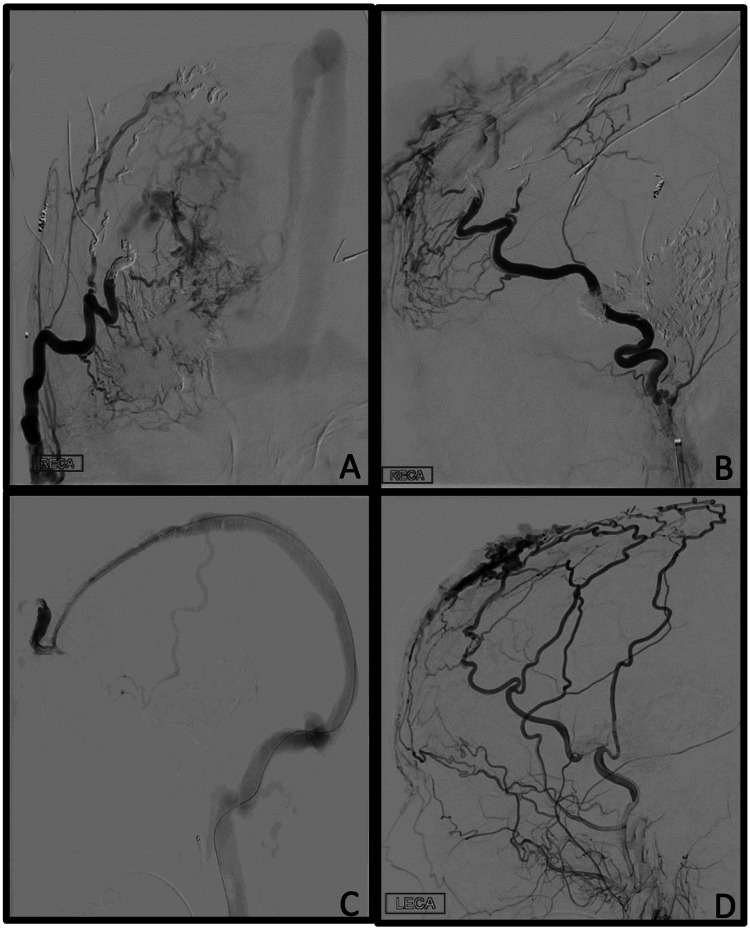
Transarterial and transvenous embolization of scalp AVM DSA status posts transarterial coil embolization with AP (A) and lateral (B) projections of a selective right external carotid artery injection. There is successful coil placement into multiple arterial feeders of the sAVM. There is a significant reduction in arterial supply to the sAVM post embolization; however, the persistent multiple small scalp branches result in the delayed filling of the sAVM. Lateral (C) projection of a selective transvenous approach to fistulous pouch accessed via sinus pericranii for coil embolization. DSA lateral projection of external carotid artery injection (D) s/p transvenous embolization. There is successful coil embolization of the posterior aspect of the sAVM proximal to the multiple drainage pathways of the sAVM and distal to the common drainage of the arterial feeders into venous varix. There is decreased opacification of the arteriovenous malformation. There is a significant reduction in overall size and arterial supply to the sAVM post embolization; however, collateral flow from the internal maxillary and posterior superficial temporal artery branches provides arterial feeders to the sAVM. sAVM: scalp arteriovenous malformation

Given the nature of her neurological morbidity and complex quartet of vascular malformations, treatment was focused primarily on the sAVM with a staged embolization involving both transarterial and transvenous approaches. A right-sided transfemoral artery embolization successfully deposited coils into five feeding branches of the right external carotid artery supplying the sAVM, namely from distal branches of the superficial temporal artery (Figure 4). Next, a left-sided transvenous embolization deposited additional coils into the common venous pouch accessed through the transosseous sinus pericranii via the SSS. This dramatically reduced the size and filling of the large sAVM (Figure 4).

Serial endovascular treatments of her sAVM led to a significant reduction in size and pulsatility. Moreover, the pain from the lesions regarding tenderness to palpation was concurrently decreased. We ultimately discussed surgical resection along with the aid of facial plastic surgery. Despite multiple treatments, serial angiographic evaluations of her scalp lesion showed persistent new development of collaterals. Ultimately the patient elected to forego further treatment for her sAVM.

## Discussion

To our knowledge, there has yet to be another report of a single patient with a quartet of vascular malformations within the literature. Scalp AVMs, on their own, are rare extracranial homologs of their more commonly encountered intracranial counterparts. Thus far, congenital, traumatic, iatrogenic, and physiological etiologies (pregnancy, hormonal hypersecretion, etc.) are described, although predominantly within the pediatric and adolescent population [1-4,7-13]. The spectrum of presentation in the adult population, natural history, and treatment options that minimize recurrence risk and maximize symptom resolution requires further validation. 

Genetic syndromes predisposing individuals to multiple vascular malformations include ataxia telangiectasia, von Hippel-Lindau syndrome, and hereditary hemorrhagic telangiectasia [6,8,14,15]. On the contrary, Wyburn-Mason syndrome is a non-hereditary subcomponent of the craniofacial arteriovenous metameric syndrome (CAMS, including the Sturge-Weber syndrome) presenting with unilateral AVMs along the optic tract and within the brain parenchyma. However, Wyburn-Mason syndrome obeys an embryonic distribution restrictive to the prosencephalon or rhombencephalon and still only describes multiple AVMs occurring within a single patient. The capillary malformation-AVM syndrome (CM-AVM) presents with cutaneous capillary malformations and AVMs of the brain and spine due to an autosomal dominant mutation in ephrin B (EPHB4) and the G-protein Ras (RASA1) [5,6]. A lack of other notable findings on examination suggests, in our case, a non-syndromic etiology. The development of multiple distinct vascular malformations, such as in our patient's case, most likely has an underlying genetic component that provided a predisposition to vascular malformation development. However, we postulate that this rare finding also builds from previous reports of altered hemodynamic flow parameters in response to an index pathology that stimulates a sequential and progressive pathway of lesion development. 

A network of neoangiogenic factors and pathways critical for vessel remodeling have progressively been uncovered by studying human cerebrovascular tissue. A non-exhaustive list, including procollagen-lysin, 2-oxoglutarate 5-dioxygenase 2 (PLOD), KRASG12V, Ras/MAPK, Wnt, BMP/TGF- β, and Sry-box 2 (SOX2), likely contribute to de novo vascular malformations in the carotid circulation [6-9,11,14-17]. A mosaic assortment of these genetic pathways and other potential somatic mutations may contribute to our patient's clinical findings. However, in addition to genetic factors, our patient's case likely supports a previously described hypothesis on flow dynamic adaptations to venous hypertension [4,7-9,11,15]. Lanzino et al. described a case of a 4-year-old boy with a midline sAVM draining into the SSS alongside a large intraparenchymal AVM. They propose that the intraparenchymal AVM was the index lesion and draw attention to retrograde filling of the SSS from the intraparenchymal AVM as evidence of venous hypertension within the dural sinuses. This subsequent communication of dural venous pressure to scalp veins will encourage venous hypertrophy, engorgement, and subsequent neoangiogenesis.

This invariably generates an ischemic state that promotes discordant angiogenesis of the existing vasculature to form de novo shunts that target nearby dural sinuses. Chronicity of both lesions can result in an enlargement of the scalp AVM to a point. Furthermore, if venous hypertension persists, it may stimulate the formation of another malformation, such as the dAVF noted in our patient. It represents another high-capacitance, low-resistance outlet, particularly when considering the sinus pericranii [4]. A quartet of vascular malformations likely is multifactorial in etiology secondary to an underlying genetic predisposition and developmental neurophysiological changes that make early diagnosis difficult. 

 Scalp AVMs have historically received varied treatments based on patient and lesion characteristics. Chowdury et al. review their institutional experience with a series of eight patients with sAVM. After triaging these lesions as either high-flow or low-flow via multimodal imaging, all patients underwent surgical resection with an average follow-up of 3.6 years. With an age range from 4 - 72 years, and male predominance, the majority experienced lesional enlargement in the third decade of life, and 3/8 had previously experienced recurrent hemorrhage. With most of their patients having high-flow lesions, they state that surgical treatment offers the most definitive hemostatic and cosmetic result due to the ability to manage multiple arterial feeders supplying the nidus and better control of the tissue for wound healing [1]. Other groups, such as Lanzino et al., and Bekelis et al., similarly report success with surgical scalp AVM resection alone [4,13]. Other authors describe in detail the use of embolization as a robust adjuvant to surgery [2,18]. A review by Gupta et al. highlights scattered reports demonstrating success with options such as percutaneous direct puncture coil embolization, endovascular coil embolization, and transarterial embolization with sclerosant [3].

Given the high baseline vascularity of this complex sAVM with sinus pericranii in tandem with the bAVM and dAVF, surgery was not considered the initial therapy. Coil embolization alone was insufficient to prevent previously angiographically occult feeders from arising. Transvenous-trans-sinus pericranii coil embolization successfully reduced the flow through the sinus pericrania and, subsequently, the sAVM nidus by eliminating a significant source of hypertensive venous outflow. Although our patient refused further intervention, we still recommend prioritizing definitive treatment options whenever possible, such as surgical resection or sclerotherapy as an adjuvant to endovascular therapy, especially in the case of a large sAVM in tandem with other vascular malformations. An isolated endovascular approach may limit patient morbidity secondary to a long treatment course.

## Conclusions

We provide the first report of an adult patient with four distinct vascular malformations involving three separate compartments - a persistent scalp AVM with sinus pericranii, an inoperable intracranial SM-V bAVM, and a Cognard I dAVF. Treatment paradigms for sAVMs are limited to case reports and small series; however, we purport that the most successful therapeutic approaches are multimodal and likely should incorporate surgical resection when feasible. We emphasize the caution required for patients with multiple other underlying intracranial vascular malformations. We believe the altered intracranial flow dynamics can drastically hinder the success of a unimodal approach involving endovascular therapy alone.
